# Histopathological changes in the electrical conduction of cardiac nodes after acute myocardial infarction in dogs and horses, compared with findings in humans: A histological, morphometrical, and immunohistochemical study

**DOI:** 10.14202/vetworld.2023.2173-2185

**Published:** 2023-10-25

**Authors:** Fabián Gómez-Torres, Luis Ballesteros-Acuña, Amparo Ruíz-Sauri

**Affiliations:** 1 Department of Basic Sciences, School of Medicine, Universidad Industrial de Santander, Bucaramanga, Colombia; 2 Department of Pathology, Faculty of Medicine, Universitat de Valencia, Valencia, Spain; 3 INCLIVA Biomedical Research Institute, Valencia, Spain

**Keywords:** atrioventricular node, dog, horse, myocardial infarction, sinoatrial node

## Abstract

**Background and Aim:**

The heart conduction system is responsible for the occurrence of various types of cardiac arrhythmia. This study aimed to histologically and morphometrically describe damaged cardiac nodes during acute myocardial infarction and to compare them with normal tissues in dogs and horses.

**Materials and Methods:**

This study describes the morphometry of cardiac nodes in five dogs and five elderly horses that succumbed to sudden cardiac death (SCD). A computerized morphometric study was conducted to determine the number of cells composing the nodes, different shape and size parameters of nodes, and their relationship with degenerative changes due to cardiac conditions.

**Results:**

In both species, the sinoatrial node (SAN) was ovoid in shape whereas the atrioventricular node (AVN) was pyramidal in shape. The percentage of collagen fibers inside the SAN of dogs (47%) and horses (50%) was found to be higher than that of cells. In contrast, the percentage of cells in the AVN of dogs (24%) and horses (16%) was higher than that of connective tissues. In the SAN, the area (p = 0.09), maximum diameter (<0.001), and mean diameter (0.003) of P cells were larger in dogs than in horses.

**Conclusion:**

Overall, the SAN cells and surrounding cardiomyocytes in dogs and horses as well as the AVN cells in dogs that succumbed to SCD decreased in size compared with those in normal hearts.

## Introduction

In humans, the most obvious research finding on the natural development of coronary heart disease is the high incidence of sudden cardiac death (SCD) [1–3]. The results of pathological studies not only supported the prevalence of severe coronary atherosclerosis in SCD but also indicated that 10%–50% of victims suffered an asymptomatic acute myocardial infarction (AMI) [4] and a higher percentage suffered subclinical early myocardial ischemia [5]. Similar findings obtained in other species can be attributed to the similarities in cardiac function between humans and these species, especially dogs [6]. The sinoatrial node (SAN) initiates the cardiac electrical impulse, which controls the coordinated rhythmic contractions of the mammalian heart [7–11]. It initiates heartbeat through pacemaker cells, which generate spontaneous electrical signals and specialized conduction pathways, carrying electrical impulses from the pacemaker cells to the adjacent atrial tissue [10, 12, 13]. Abnormal SAN morphology or function can inappropriately accelerate or slow down the heart rate, leading to fatal cardiac arrhythmia. This condition puts humans and animals at risk for heart diseases, such as atrial fibrillation (AF) and heart failure (HF), which in turn can cause syncope and SCD [1, 14]. Sick sinus syndrome (SSS) is a common bradyarrhythmia that occurs in both humans and dogs; it is the second most common indication for permanent artificial pacemaker implantation in canines [15–18]. In clinical practice, SSS is often diagnosed based on abnormal SAN activity on an electrocardiogram, with corresponding clinical signs of low cardiac output (syncope, staggering, and weakness) [19]. The main electrocardiographic findings in SSS are sinus arrhythmia and bradycardia, periods of sinus arrest, and/or paroxysmal atrial tachycardia alternating with bradycardia (commonly called bradycardia–tachycardia syndrome) [1].

Cardiac conditions are suspected to cause SCD or collapse in racehorses [20]. Sudden cardiac death in racehorses resembles SCD in young adult humans (below 35-years-old), with the latter frequently occurring during physical activity [21, 22]. In a study on racehorses that succumbed to SCD, fibrotic lesions were clearly observed in the atrium in the vicinity of the SAN. Severe fibrosis in areas adjacent to the node compared with normal SAN evidenced a decrease in SAN fibers. Histological findings in SCD include foci of myocardial fibrosis located in the right atrium, interatrial septum, and upper portion of the interventricular septum, including the atrioventricular conduction system [23–25]. Humans and dogs have quite similar atrioventricular node (AVN) structures [2, 26]. Dual AVN conduction is a commonly observed electrophysiological response in both species [27, 28]. However, AVN re-entrant tachycardia is the most common form of supraventricular tachyarrhythmia in humans, whereas it has been reported only once in dogs [29–31] for as yet unclear reasons. Whether the AVN re-entrant tachycardia circuit is confined to the node or involves part of the perinodal atrial tissues remains unclear [32]. Studies on surgical and catheter ablation support the concept that extranodal atrial tissues are partially involved in the re-entry circuit [33]. A complete atrioventricular block occurs when a cardiac impulse is completely and permanently interrupted in the AVN, atrioventricular bundle (AVB), or branches. The atria are normally depolarized by the SAN, but the ventricles are depolarized by subsidiary pacemakers below the block area, where the discharge rate of the electrical impulse is reduced [34]. Studies have described marked pathological changes in horses that succumbed to SCD, such as focal myocardial fibrosis and ischemia in the atrium as well as in the atrioventricular junction and upper interventricular septum, including the AV conduction system (AVN, AVB, and left and right bundle branches). Furthermore, a fibroblastic response has been observed in this connective tissue-rich area [10, 23, 24]. Supraventricular arrhythmia is one of the main causes of morbidity and mortality among animal species.

This study aimed to histologically and morphometrically describe damaged cardiac nodes during AMI and to compare them with normal tissues in dogs and horses. Lesions in the cardiac nodes of these animal species were also compared with the lesions found in humans in existing studies.

## Materials and Methods

### Ethical approval

Procedures were conducted in accordance with the Ethics Committee of the Universidad Cooperativa de Colombia (No. 014-2018) and in compliance with Law 84 of 1989 at the national level, corresponding to the use of animals in experimentation and research, Chapter VI of the “National Statute for the Protection of Animals.”

### Study period and location

This study was conducted between November 2021 and November 2022 in the city of Bucaramanga, Colombia.

### Sample processing

Hearts of 10 male horses (weighing 250–300 kg and aged 6–8 years) with a history of heart disease were obtained from necropsies performed by clinical veterinarians. In addition, the hearts of 10 male dogs (adult medium-sized breed animals weighing 8–19 kg and aged 10–14 years) with a history of cardiac pathologies (myxomatous valvular degeneration and Grade 3–6 heart murmur), documented by electrocardiography and echocardiography, were obtained from necropsies performed by clinical veterinarians. The hearts of 10 male horses destined for slaughter (weighing 250–300 kg and aged 2.5–3.5 years) and the hearts of 10 dogs (adult medium-sized breed animals weighing 8–19 kg and aged 5–12 years) without a history of heart disease were used for comparison with the hearts of animals that died from heart disease.

### Sinoatrial node

In both horses and dogs, the SAN region was sectioned at the junction of the cranial vena cava with the right atrium, in which the SAN lies directly beneath the sulcus terminalis and the anterior margin is near the junction of the right atrial appendage (auricle) with the superior vena cava, which is marked by a small epicardial ridge (crista terminalis). The SAN was subsequently cut into 5-mm-thick slices.

### Atrioventricular node

The AVN is a few millimeters anterior to the orifice of the coronary sinus. The node is located below the endocardium of the right atrial septum and just above the septal junction of the tricuspid valve. The AVN region was serially sectioned, and the tissue block removed for the study included the entire junction of the interatrial and interventricular septum. Sections were cut perpendicular to the line of junction of the septum, obtaining approximately six samples per node. All samples were fixed in 5% formaldehyde, labeled with numbers, and embedded in paraffin. The sections were stained with Hematoxylin-Eosin and Masson’s trichrome, and histological samples were cut into 5-μm-thick sections using a microtome.

### Image analysis and histopathological evaluation

#### Node morphometry

Samples were evaluated using a Leica DMD108 light microscope (Leica Microsystems, Wetzlar, Germany). For morphometric analysis, 220 micrographs of tissue from the SANs and AVNs were taken at different magnifications: 4× to measure the node parameters and 10× or 20× to measure the cell parameters The area, mean density, maximum and mínimum diameters, perimeter, roundness, length, and width of the nodes were measured at 4× magnification. For roundness, a morphometric measurement value far from 1 indicates an elongated or irregular contour, whereas values close to 1 indicate a rounder cell or node.

#### Cellular morphometry

Inside each node, we quantified the area, maximum and minimum diameters, mean diameter, and roundness of P cells, T cells, and peripheral cardiomyocytes (50 cells were evaluated for each cell type and each case). Immunohistochemical staining with Anti-Human Desmin Clone D33-IR606 (Dako Corporation. Santa Clara, United States) was performed to visualize the desmin intermediate myofilaments localized in the node cells. The myofilaments were compared with the surrounding cardiomyocytes. A computerized morphometric study was conducted using Image-Pro Plus 7.1 (Media Cybernetics, Silver Spring, MD, USA). After the manual selection of each micrograph based on color histograms, they were calibrated and the total tissue area (mm^2^) was obtained. Furthermore, in the SAN and AVN, we determined the percentage of the components of each node by segmentation (the morphometric program determines the components only in percentage), in which red denotes the percentage of collagen fibers; green, percentage of cells; and yellow, percentage of fundamental substance or adipose tissue.

### Statistical analysis

Descriptive statistics, graphical representations, and hypothesis testing were conducted using statistical package for the social sciences (SPSS) 20 (SPSS, Chicago, IL, USA) and Microsoft Excel 2013. p < 0.05 was considered to indicate statistical significance. The confidence interval for continuous variables was 95%. The Kolmogorov–Smirnov normality test was conducted for each morphometric parameter, and descriptive statistics were calculated also for each parameter. To ensure statistical robustness, the Mann–Whitney U test was employed for quantitative variables, variables with non-normal distribution, and comparison of cells in the two species. For normal variables, such as node parameters, student’s t-test was used. Data were expressed as mean ± standard deviation for all measured lengths.

## Results

### Sinoatrial node

The SAN is located at the junction of the cranial vena cava and free wall of the right atrium, where it lies directly beneath the sulcus terminalis. The arterial branch, which irrigates the SAN in dogs, is located at the substance of the node in dogs, whereas in horses, it is located at one of its poles.

**Figure-1:**
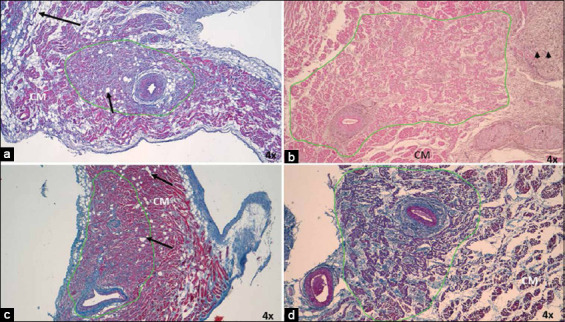
Histology of cardiac nodes in dogs and horses with acute myocardial infarction at 4×. (a) Sinoatrial node (SAN) in dogs with hematoxylin-eosin and (c) Masson’s trichrome and (b) in horses with hematoxylin-eosin and (d) Masson’s trichrome. Note the infiltration of fatty tissue (arrows) at the periphery and interior of the node in dogs, which replace the cardiomyocytes and nodal cells, respectively. Increased fibrous tissue at the periphery of the SAN in horses (arrowheads). CM=Cardiomyocytes. 4× magnification.

In the infarcted dogs and horses, the SAN presented an ovoid shape (Figure-1), with no differences observed relative to control cases. A thin capsule of connective tissue was found surrounding the node in all horses. The different morphometric parameters measured for the SAN of both species are presented in Table-1 [6].

**Table-1:** Summary of parameters measured in the sinoatrial and atrioventricular nodes in dogs and horses with infarction, in comparison with reported normal values.

Parameter	Infarcted dogs. µm (SD)	Infarcted horses. µm (SD)	p-value	Normal dogs. µm (SD) [6]	Normal horses. µm (SD) [6]
Sinoatrial node					
Area	208,1830 ± 573,682	2,933,089 ± 1,234,124.32	0.276	1,090,064.65 ± 600,507.09	2,083,094.36 ± 810,250.48
Mean density	0.21 ± 0.05	0.29 ± 0.19	0.208	0.17 ± 0.04	0.24 ± 0.07
Max. diameter	2,466.39 ± 386.59	2,755.13 ± 1,874.23	0.522	1,704.55 ± 610.55	2,065.99 ± 305.85
Min. diameter	1,037.11 ± 231.87	1,396.40 ± 801.2	0.260	684.48 ± 54.08	1,252.29 ± 331.21
Perimeter	6,345.58 ± 905.69	7,164.76 ± 5,487.6	0.478	4,374.18 ± 1,645.36	5,544.59 ± 836.32
Roundness	1.58 ± 0.31	1.39 ± 1.15	0.633	1.41 ± 0.31	1.27 ± 0.24
Atrioventricular node					
Area	480,598.14 ± 158,269.59	4,846,972.33 ± 1,874,349	0.001	740,939.48 ± 819,443.39	1,149,812.05 ± 919,697.71
Mean density	0.24 ± 0.06	0.18 ± 0.16	0.178	0.33 ± 0.14	0.21 ± 0.07
Max. diameter	1,160.30 ± 172.36	2,877.36 ± 1,654.57	0.006	1,386.14 ± 397.80	1,903.49 ± 650.87
Min. diameter	512.07 ± 181.77	1,649 ± 1,238.43	0.001	514.16 ± 416.18	660.10 ± 317.48
Perimeter	3,239.81 ± 518.99	8,409.45 ± 3,231.27	0.002	3,813.48 ± 1,514.01	4,634.11 ± 1,407.81
Roundness	1.79 ± 0.36	1.48 ± 1.11	0.212	2.30 ± 0.97	1.76 ± 0.42

Areas were measured in μm^2^. Roundness has no units of measure. SD=Standard deviation

**Figure-2:**
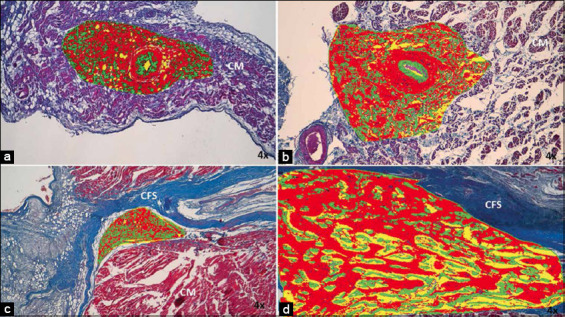
Morphometric analysis of sinoatrial node (SAN) in (a) dogs and (b) horses and (c) atrioventricular node in dogs and (d) horses infarcted at 4×. Note the large number of collagen fibers inside the heart nodes in both species, which is represented by red, and the decrease in cells represented by green. Yellow indicates the fundamental substance of the heart nodes, but in the SAN of dogs it is replaced by adipose tissue. CM=Cardiomyocytes, CFS: Cardiac fibrous skeleton. 4× magnification.

In dogs, the main changes were observed in nodal cells (P and T cells) and cardiomyocytes surrounding the node. We observed hypertrophy of P cells in infarcted dogs as these cells were larger than those in control cases. The P and T cells were easily detected as they were paler than the surrounding cardiomyocytes. Adipose tissue replacing the fundamental substance and cells was observed inside the node (Figures-1a and 2a). Furthermore, in 70% of infarcted dogs (Figures-1a and c), we observed a decrease in the area occupied by cardiomyocytes in the periphery of the node, which comprised adipose tissue in the atrial myocardium. Another important finding was the higher percentage of collagen fibers (fibrosis) inside the SAN (47%) than the percentage of cells inside the node (33%), which was not observed in our control cases (Table-2 and Figure-2a) [35].

**Table-2:** Structural components of cardiac nodes expressed as percentages in dog and horse infarcted and normal hearts.

Species	Dogs				Horses			
		
Node	Infarcted SAN	Infarcted AVN	Normal SAN	Normal AVN [35]	Infarcted SAN	Infarcted AVN	Normal SAN	Normal AVN [35]
% collagen fibers	47	48	34.2	9.75	50	44	43.3	37.24
% fundamental substance		28	22.9	63.98	19	40	6.3	11.2
% cells	33	24	43	26.27	31	16	50.4	51.56
% adipose tissue	20							

**Figure-3:**
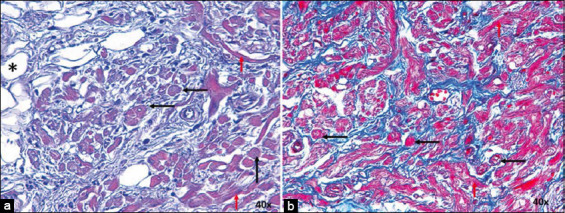
(a) Sinoatrial node from infarcted dogs stained with Hematoxylin-Eosin and (b) Masson’s trichrome at 40×. P cells are indicated by black arrows and T cells by red arrows. Note the presence of adipose tissue inside the node (*). CM=Cardiomyocytes. 20× magnification.

**Figure-4:**
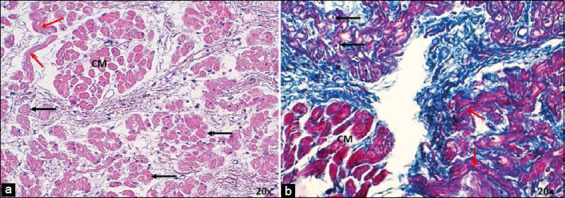
(a) Sinoatrial node from infarcted horses stained with hematoxylin-eosin and (b) Masson’s trichrome at 20×. P cells are indicated by black arrows and T cells by red arrows. CM=Cardiomyocytes. 20× and 10× magnification.

The infarcted horses had smaller P and T cells than those with normal hearts (Figures-3 and 4). In these species, we also observed a moderate presence of fibrosis in the atrial myocardium surrounding the SAN (Figure-1b and d). Another important finding was the higher percentage of collagen fibers (fibrosis) inside the SAN (50%) than the percentage of cells inside the node (31%), which was not observed in our control cases (Table-2 and Figure-2b).

Table-3 presents the different parameters measured in nodal cells, such as area, diameter, and roundness. No statistically significant differences were observed between the morphometric parameters measured in the SAN of both dogs and horses. In P cells, the area (p = 0.09), maximum diameter (p < 0.001), and mean diameter (p = 0.003) were larger in dogs than in horses. These cells were rounder in horses than in dogs, with a value closer to 1 (p = 0.007). Only the maximum diameter of T cells was greater in dogs than in horses (p = 0.02). The dogs had more elongated cell shape, with a value quite far from 1 (p = 0.19). No significant between-species differences were observed in cardiomyocytes.

**Table-3:** Overall summary of morphometric parameters of P cells, T cells, and cardiomyocytes in infarcts from sinoatrial and atrioventricular nodes in dogs and horses, compared with reported normal values.

Species	Cell	Node	Area. µm^2^(SD)		Max. diameter µm (SD)		Min. diameter µm (SD)		Mean diameter µm (SD)		Roundness (SD)	
				
			I	N	I	N	I	N	I	N	I	N
Dog	P cell	SAN	174.97 ± 31.73	688 ± 71.03	18.19 ± 2.94	42.47 ± 8.74	11.73 ± 1.26	19.82 ± 1.76	14.53 ± 1.38	29.40 ± 1.57	1.23 ± 0.27	1.40 ± 0.27
		AVN	101.42 ± 26.70	353.83 ± 66.3	12.77 ± 1.63	24.35 ± 2.53	9.07 ± 1.91	17.08 ± 1.82	10.95 ± 1.33	20.58 ± 2.18	1.09 ± 0.14	1.10 ± 0.01
Horse		SAN	136.21 ± 46.20	976 ± 223.7	14.15 ± 2.70	39.87 ± 6.44	10.91 ± 1.89	29.75 ± 5.01	12.48 ± 2.17	34.47 ± 4.14	1.06 ± 0.05	1.14 ± 0.07
		AVN	1.684.75 ± 739.27	618.21 ± 80.1	56.10 ± 15.54	32.37 ± 2.67	35.03 ± 8.78	22.85 ± 2.12	43.90 ± 10.45	27.65 ± 1.85	1.21 ± 0.07	1.13 ± 0.02
Dog	T cell	SAN	205.24 ± 60.13	599.8 ± 194	30.71 ± 7.31	65.26 ± 12.20	7.65 ± 1.84	11.36 ± 3.11	16.07 ± 2.22	29.16 ± 2.77	2.06 ± 0.53	3.04 ± 1.15
		AVN	138.32 ± 36.44	204.94 ± 85.3	19.67 ± 2.67	27.03 ± 10.51	8.04 ± 1.58	9.14 ± 1.72	13.12 ± 1.46	16.99 ± 5.29	1.37 ± 0.14	1.66 ± 0.39
Horse		SAN	174.67 ± 49.98	1.090 ± 257	24.50 ± 5.21	71.04 ± 11.13	8.60 ± 1.55	19.17 ± 2.36	15.43 ± 3	35.73 ± 3.87	1.59 ± 0.39	1.98 ± 0.16
		AVN	3.927.29 ± 771.50	438.66 ± 82.3	135.51 ± 21.81	47.47 ± 6.03	30.99 ± 5.89	10.71 ± 1.90	62.93 ± 5.85	27.34 ± 1. 0.55	2.19 ± 0.47	2.26 ± 0.39
Dog	Cardiomyocyte	SAN	153.35 ± 36.06	519.1 ± 163	15.03 ± 2.10	29.70 ± 8.39	11.64 ± 1.67	20.66 ± 3.32	13.31 ± 1.69	25.13 ± 4.44	1.07 ± 0.02	1.15 ± 0.10
		AVN	115.66 ± 30.17	287.32 ± 69.7	13.73 ± 3.27	20.46 ± 1.87	9.62 ± 1.67	16.62 ± 2.79	11.70 ± 1.75	18.39 ± 2.37	1.09 ± 0.17	1.08 ± 0.02
Horse		SAN	139.10 ± 35.19	529.3 ± 165	14.53 ± 1.82	29.69 ± 4.60	10.91 ± 1.87	20.97 ± 3.78	12.73 ± 1.64	25.36 ± 3.93	1.07 ± 0.03	1.13 ± 0.02
		AVN	386.71 ± 142.80	294 ± 104.9	24.13 ± 4.62	21.49 ± 4.25	18.82 ± 3.91	16.06 ± 3.45	21.31 ± 4.15	18.48 ± 3.46	1.09 ± 0.03	1.09 ± 0.05
Scheme			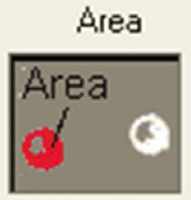		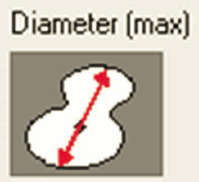		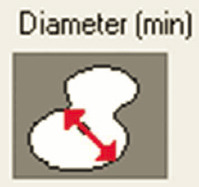		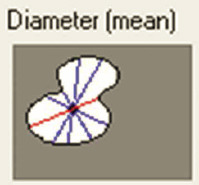		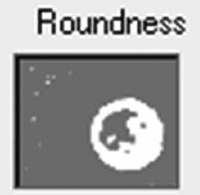	

Areas were measured in μm^2^ . Roundness has no units of measure. I=Infarcted. N=Normal [35]. SD=Standard deviation, SAN=Sinoatrial node, AVN=Atrioventricular node

### Atrioventricular node

In mammals, the AVN is located subendocardially in the right half of the interatrial septum. It can be seen anterior to the ostium of the coronary sinus and above the junction of the septal junction of the tricuspid valve. However, the arterial branch of the AVN that supplies it could not be identified in dogs or horses.

**Figure-5:**
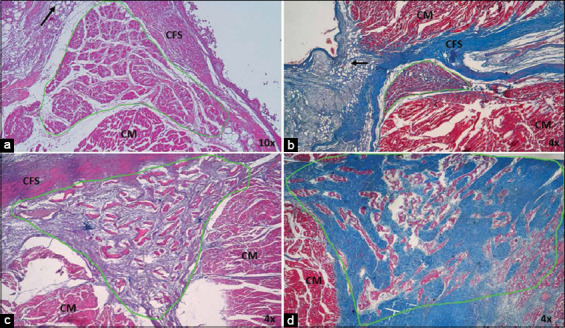
(a) Histology of infarcted atrioventricular node (AVN) from dogs stained with Hematoxylin-Eosin at 10× and (b) Masson’s trichrome at 4× and (c) AVN from horses stained with Hematoxylin-Eosin and (d) Masson’s trichrome at 4×. Note the adipose tissue infiltrating the cardiac fibrous skeleton (CFS) and periphery of the node, replacing cardiomocytes (CM) in dogs (arrows). 4× magnification.

**Figure-6:**
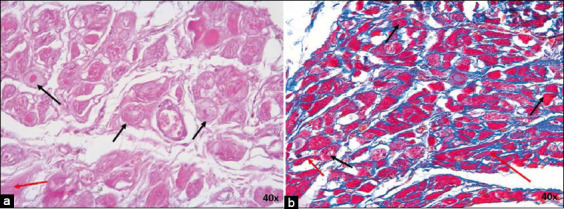
(a) Atrioventricular node from infarcted dogs stained with Hematoxylin-Eosin and (b) Masson’s trichrome at 40×. P cells are indicated by black arrows: note the prominent nucleoli in some of these cells. T cells are indicated by red arrows. CM=Cardiomyocytes; CFS=Cardiac fibrous skeleton. 20× magnification.

AVN presented a pyramid shape in both species. The parameters obtained by morphometry at this node in both species are presented in Table-1. In infarcted dogs, a decreased percentage of cells was observed in compared with control cases (24%), and an increase in the percentage of collagen fibers (fibrosis) (Table-2 and Figure-2c). An increase in the percentage of adipose tissue was observed in the periphery of the AVN, located particularly in the cardiac fibrous skeleton (CFS) (Figures-5a and b). The P cells were paler than cardiomyocytes, characterized by prominent nucleoli, and appeared hypertrophic. Only a few T cells were observed in the analyzed nodes (Figure-6).

**Figure-7:**
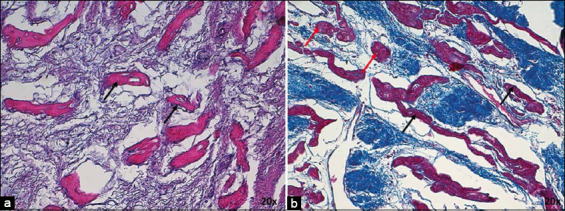
(a) Atrioventricular node of infarcted horse stained with Hematoxylin-Eosin and (b) Masson’s trichrome at 20×. P cells are indicated by red arrows and T cells are indicated by black arrows. Note the large size of the cells, the decrease in P cells, and the increase in collagen fibers within the node. 20× magnification.

In infarcted horses, we observed a decrease in nodal cells (Figures-5c and d) and an enhanced number of collagen fibers compared with control cases (Table-2 and Figure-2d). These cells appeared hypertrophic and were paler than cardiomyocytes. A substantial increase in T cells and a decrease in P cells were also noted (Figure-7). Table-3 presents the different morphometric parameters measured in nodal cells.

Comparing the two species, the area (p = 0.001), maximum diameter (p = 0.006), minimum diameter (p = 0.001), and perimeter (p = 0.002) of the SAN were greater in infarcted horses than in infarcted dogs. For the AVN, the length (2.9 ± 1.2 mm) and width (1.8 ± 0.7 mm) were greater in horses than in dogs (1.1 ± 0.1 mm and 0.6 ± 0.2 mm, respectively) (p = 0.013 for length and p = 0.009 for width).

In subsequent cell analysis, the area and diameter (maximum, minimum, and mean) of the P cells, T cells, and cardiomyocytes were significantly greater in horses than in dogs (all p < 0.001). The P cells were rounder in dogs than in horses (p = 0.001), and the T cells were more elongated in horses than in dogs (p < 0.001).

### Immunohistochemistry

**Figure-8:**
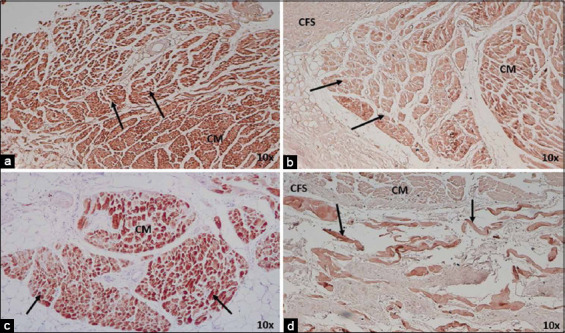
Identification of sinoatrial node (SAN) cells in (a) dogs and (c) horses and (b) atrioventricular node (AVN) cells in dogs and (d) horses by desmin immunohistochemical staining in infarcted animals at 10×. Note the difficulty in distinguishing nodal cells from cardiomyocytes in hearts that have suffered myocardial infarction in SAN from both species and in AVN from dogs, observing a higher intensity difference between nodal cells (arrows) and cardiomyocytes (CM). CFS=Cardiac fibrous skeleton. 10× and 4× magnification.

We employed desmin staining to easily identify SAN and AVN cells in infarcted dogs. We found that nodal cells exhibited high reactivity to immunohistochemical staining, but no differences were observed with cardiomyocytes, which reacted in the same manner (Figures-8a and b). In infarcted horses, nodal cells exhibited high desmin positivity in both nodes; however, in the SAN, no difference was observed with cardiomyocytes, whereas in the AVN, nodal cell positivity was higher than that in working cardiac cells (Figures-8c and d).

### Imaging findings

**Figure-9:**
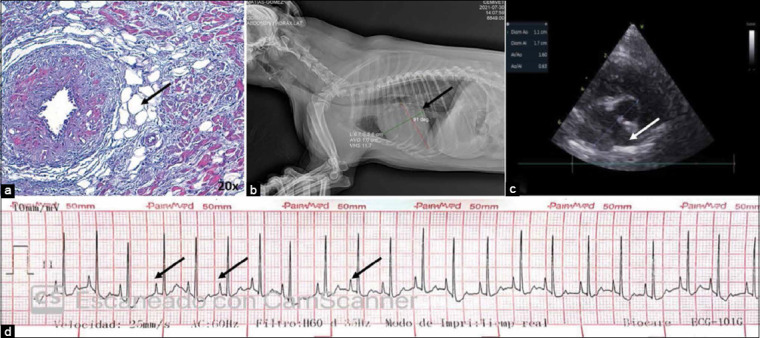
Cardiac alterations in diagnostic images compatible with changes in the electrical system in dogs. (a) Sinoatrial node with fatty infiltrate inside (arrow) at 20×. (b) X-ray showing the presence of cardiomegaly (arrow). (c) Echocardiogram with dilatation of the left atrium (arrow). (d) Electrocardiogram showing the presence of peaked P waves (arrows).

Tachycardia and different degrees of heart murmur were detected in infarcted dogs in this study, with the presence of peaked P waves on the electrocardiogram, indicating a type of supraventricular arrhythmia (paroxysmal atrial tachycardia). Furthermore, different grades of left atriomegaly were found on the imaging tests. Generalized cardiomegaly and cardiosternal contact of the entire right ventricular surface were also observed on radiography. These findings are compatible with changes in the electrical conduction system, as observed in our study’s histological and morphometric evaluation (Figure-9).

## Discussion

Given the critical role the SAN and AVN play in activating and coordinating cardiac contraction as well as their involvement in inducing arrhythmias, this study aimed to describe the histological and morphometrical alterations in cardiac nodes after AMI in both horses and dogs. The ovoid shape of SAN has been described in dogs with nodal heart disease and animals without a history of heart disease [36, 37].

The morphometric parameters measured in the SAN, such as diameter, area, perimeter, density, and roundness, were greater in this study of animals that died of myocardial infarction than in a previous study by Gómez-Torres *et al*. [6] of dogs without a history of cardiac events. The difference in parameters was caused by a decrease in the noble parenchyma and the growth of connective tissue (fibrosis) inside the node. We observed a significant increase in collagen fibers and adipose tissue in infarcted dogs, replacing fundamental substance and cells, which was unusual and not observed in healthy hearts. Such an increase in collagen fibers (fibrosis) can lead to SAN dysfunction.

Morphologic and functional abnormalities in the SAN can cause the heart rate to increase or decrease, resulting in fatal cardiac arrhythmias. The abnormalities in the SAN also increases the risk of AF or HF, which can cause syncope and SCD [1, 15]. Another cause of this SAN malfunction is SSS, which is defined by symptomatic dysfunction of this node with an inadequate atrial rate for physiological requirements [38]. Sick sinus syndrome has been reported in humans and dogs [18, 37] and is the second most common indication for permanent artificial pacemaker implantation in canines [15, 16]. It is caused by abnormalities in SAN automaticity and/or sinoatrial conduction, which can be multifactorial [38]. The most common cause of intrinsic changes in the SAN is age-related, idiopathic degenerative fibrosis of the nodal tissue [39]. This is consistent with our findings, as the increase in collagen fibers (fibrosis) observed may be related to SSS in the dogs that died of myocardial infarction at an advanced age in this study.

The presence of arrhythmias common to dogs and humans has been reported in SSS, where the presence of tachybradycardia, bradycardia, prolonged atrial pauses, and SAN exit and re-entry blocks was reported in dogs experimental models of myocardial infarction [40, 41]. These SAN alterations indicate the similarity that exists between the two species and highlights the importance of our results, which can thus be applied to human medicine.

In dogs with SSS, extensive SAN destruction has been reported, characterized by decreasing numbers of nodal cells replaced by adipose or fibroadipose tissue and interrupted contiguity between the node and atrial myocardium [37, 42]. These findings are consistent with ours, indicating that adipose tissue was found inside the node replacing fundamental substance and cells. Another finding was interstitial myocardial fibrosis with loss of muscle fibers in both atria [37, 42], but this was not observed in our study.

In addition to other intrinsic cellular features, the increased presence of collagen fibers in the hearts of larger species has been suggested to play a role in decreasing heart rate. Between 20% and 30% of collagen fibers have been reported inside the SAN of healthy dogs [43], along similar lines to the 34% found in healthy dogs in our study; in healthy humans, this percentage is reportedly between 35% and 55%, indicating a lower heart rate than in dogs [43]. This shows that the percentage of collagen fibers significantly increased in our current study of animals with myocardial infarction, potentially leading to electrical conduction alteration through the node. This could also occur in humans due to the similarities with the dog.

In humans and experimental dogs, HF and AMI have been shown to worsen fibrosis in the SAN [40, 43], which disrupts the coupling between the conduction cells of the SAN and affects their function, leading to bradycardia, conduction blocks, and re-entry tachycardia5]. We demonstrated the worsened fibrosis in our study in dogs and horses when comparing these cases with healthy animal hearts, showing the potential damage in the electrical conduction system.

Moderate to marked fibrosis, replacement of cardiomyocytes by fatty tissue, and vascularization of the conduction system fibers have also been described in humans with AMI, mainly affecting the proximal segments of the branches of the AVB, AVN, and SAN [5]. This is consistent with the findings of our study (predominantly in dogs and to a lesser extent in horses).

Comparing SAN nodal cells in our group’s previous study by Gómez-Torre *et al*. [6] on hearts from dogs without a history of heart disease, with P cells from infarcted dogs, we found smaller areas and diameters in animals that succumbed to SCD. Furthermore, the P cells in cases that succumbed to SCD were rounder than those in normal nodes. In the same cases, the T cells had lower morphometric parameters than those from normal nodes. This condition in SAN cells can lead to any type of supraventricular arrhythmia. Analysis of the cardiomyocytes surrounding the node revealed decreases in the morphometric parameters to half the values described in normal hearts, affecting the continuity of the electrical impulse through the atrial myocardium, which leads to abnormal depolarization of the cardiomyocytes.

The alterations observed in our study, also described by different authors as aforementioned, are consistent with the imaging and clinical diagnostic findings in the dogs we evaluated. The presence of peaked P waves, which resulted in paroxysmal atrial tachycardia, was observed on electrocardiography, in addition to dilated left atria diagnosed through echocardiography and cardiomegaly via X-ray. The patients in this study exhibited tachycardia and murmurs of different degrees based on semiological evaluation during consultation. As observed in our various histological and morphometric evaluations, changes in cardiac node cell size, increased collagen fibers inside or at the periphery of the nodes, and cardiomyocyte replacement by fibrous or fatty tissue, all point to a correlation between changes in the electrical conduction system and results obtained using diagnostic imaging methods.

We observed ovoid-shaped SAN in horse hearts without apparent heart disease, similar to our findings in horses that succumbed to SCD due to AMI. A small capsule of connective tissue was detected around many of the nodes evaluated in this study, but this capsule was not observed in normal nodes.

This study found that the morphometric parameters (length, axes, diameters, perimeter, and radii) measured in the SAN of horses affected by AMI were higher than those in the nodes of healthy horses. These differences, which can trigger node malfunction, are potentially caused by an increase in the percentage of collagen fibers (fibrosis) inside the node, which is consistent with our findings in dogs.

Sick sinus syndrome can also occur in horses, but the incidence is lower than those in humans and dogs; one case of SSS in horse that occurred after collapsing during exercise was managed with a dual-chamber rate pacemaker [44]. Physiological bradyarrhythmias are common events in horses [45]. Physiological changes in the electrical impulse in the SAN can cause varying degrees of sinus arrhythmia, sinus pauses, and sinus outflow block. These cardiac rhythm disturbances are less common than AVN changes in horses [25, 46].

In horses that succumbed to SCD, marked pathological changes such as myocardial fibrosis, inflammation, and ischemia in the atrium at the periphery of the SAN have been reported [23, 24]. We observed moderate fibrosis at the periphery of the node, which is consistent with the results of the aforementioned studies.

In this study, SAN cells and cardiomyocytes in horses exhibited significantly lower morphometric parameters (area and diameters) than those in our previous study by Gómez-Torres *et al*. [6] on normal hearts. Altogether, this points to SAN rhythm failure abnormalities as the potential cause of SCD in these animals.

The different morphometric parameters measured in the AVN of dogs in this study (major axis, maximum diameter, perimeter, maximum radius, length, and width) had lower values than those in our previous study by Torres *et al*. [6] on hearts without a history of heart disease. This demonstrates the damage produced at this node and the adverse implications for electrical impulse transmission in the atrioventricular zone, potentially resulting in ventricular arrhythmias due to failure in the physiological pause of the rhythm at this level.

Studies on atrioventricular conduction block in dogs have identified injuries to the upper border of the interventricular septum and CFS, with degeneration and fibrosis of the atrioventricular conduction fibers [47]. This degenerated CFS tissue is replaced by fibrous or fibroadipose tissue, as also occurs in the periphery and interior of the AVN, although the connection between the AVB and the atrial muscle is preserved [34]. In this study, we found that adipose tissue replaced the CFS tissue, which is consistent with the findings of Ward *et al*.[19].

A previous study by Lo *et al*. [32] on AVN re-entrant tachycardia reported oval nodes and AVN size of 2.4 mm × 1.9 mm, but fibrosis was absent. However, in our study of SCD, we observed a pyramid-shaped AVN.

Observing the behavior of nodal cells and surrounding cardiomyocytes in the AVN of dogs that succumbed to SCD, we found a significant decrease (almost 50%) in the values of the measured parameters in nodal cells and cardiomyocytes compared with those in dog hearts without clinical history of cardiac events in our previous study by Gómez-Torres *et al*. [6]. This suggests that in animals with some type of cardiac pathology, electrical conduction is affected at the atrioventricular level, predisposing them to ventricular arrhythmias.

In a study of the cardiopathology of SCD in horses, marked pathological changes such as fibrosis were observed at the atrioventricular junction, in the upper portion of the interventricular septum, including the AV conduction system [23, 24]. These findings agree with the findings of this study, although the degree of fibrosis observed was moderate, particularly around the AVN.

The morphometric parameters evaluated (area, axes, diameters, perimeter, radii, length, and width) in the AVN of horses that succumbed to SCD significantly increased compared with those in our previous study by Gómez-Torres *et al*. [6] on normal hearts. This suggests that node hypertrophy is associated with, cardiac events and verification through node component evaluation by segmentation showed a dramatic increase in the percentage of connective tissue (fibrosis), as did cell evaluation as described below.

In horses, the causes of persistent pathological AVN dysfunction were toxic agents, inflammation, fibrous or neoplastic infiltration, and congenital anomalies [30, 48–50].

In the AVN, the values of the morphometric parameters (area and diameters) of P and T cells in horses that succumbed to SCD were much greater than those in our previous study by Gómez-Torres *et al*. [6] on normal hearts. We observed T cell proliferation and P cell reduction, which could significantly inhibit electrical impulse transmission through the node. This interrupts the physiological pause performed by the AVN to allow the impulse to pass to the ventricles, which could lead to ventricular arrhythmias.

A greater presence of intermediate filaments has been observed in conduction system cells than in cardiomyocytes, mainly due to the presence of desmin, which is distributed throughout the entire cytoplasm of conduction cells, unlike in cardiomyocytes where it only occurs in certain specific points (intercalated and Z disks). This enables accurate identification of conduction system cells in normal hearts [35, 51, 52]. In this study of dogs and horses that succumbed to SCD, the use of desmin was not effective in identifying the cells of the heart nodes, as this immunohistochemical stain failed to exhibit differences between nodal cells and cardiomyocytes in the heart nodes of dogs or in the SAN of horses. Higher positivity for desmin was observed only in AVN cells of horses.

## Conclusion

In both dogs and horses, we observed a decrease in the number and size of P and T cells and an increase in the percentage of collagen fibers in the nodes of hearts with diseases. We noted a higher number of adipocytes both inside and outside the cardiac nodes in dogs than in horses.

The correlation between the pathological findings from diagnostic imaging and the alterations detected in the cardiac nodes in our study strengthens the hypothesis that damage is generated in the conduction system after myocardial infarction. Such a correlation also supports use as models in human cardiology.

This study is useful as a model for physiopathological and pharmacological experimentation due to the great similarities between the animal models and humans in terms of the configuration and function of the conduction system as well as the pathologies that affect it.

Given the great difficulty in obtaining samples from the referred species and the lack of authorization from the owners for the use of organs in research, we suggest that this issue be evaluated in other researches. Information about the morphohistological characteristics of the cardiac nodes in these species was improved, constituting a good contribution to comparative anatomy.

## Authors’ Contributions

FG, LBA, and ARS: Designed the study and drafted the manuscript. FG and ARS: Statistical analysis and interpreted the results. FG: Conducted the study. LBA and ARS: Reviewed and edited the manuscript. All authors have read and approved the final manuscript.

## References

[1] Janse M.J (2004). Electrophysiological changes in heart failure and their relationship to arrhythmogenesis. Cardiovasc. Res.

[2] Roberts W, Salandy S, Mandal G, Holda M.K, Tomaszewksi K.A, Gielecki J, Tubss R.S, Loukas M (2019). Across the centuries:Piecing together the anatomy of the heart. Transl. Res. Anat.

[3] Haissaguerre M, Cheniti G, Escande W, Zhao A, Hocini M, Bernus O (2019). Idiopathic ventricular fibrillation with repetitive activity inducible within the distal Purkinje system. Heart Rhythm.

[4] Liberthson R.R, Nagel E.L, Hirschman J.C, Nussenfeld S.R, Blackbourne B.D, Davis J.H (1974). Pathophysiologic observations in prehospital ventricular fibrillation and sudden cardiac death. Circulation.

[5] Lie J.T (1975). Histopathology of the conduction system in sudden death from coronary heart disease. Circulation.

[6] Gómez-Torres F.A, Ballesteros-Acuña L.E, Ruíz-Saurí A (2019). Histological and morphometric study of the components of the sinus and atrio-ventricular nodes in horses and dogs. Res. Vet. Sci.

[7] Keith A, Flack M (1907). The form and nature of the muscular connections between the primary divisions of the vertebrate heart. J. Anat. Physiol.

[8] Zichaa S, Fernández-Velasco M, Lonardo G, L'Heureux N, Nattel S (2005). Sinus node dysfunction and hyperpolarization-activated (HCN) channel subunit remodeling in a canine heart failure model. Cardiovasc. Res.

[9] Carmona-Puerta R, Lorenzo-Martínez E (2020). The normal sinus node:What we now know. Corsalud.

[10] Padala S.K, Cabrera J.A, Ellenbogen K.A (2021). Anatomy of the cardiac conduction system. Pacing Clin. Electrophysiol.

[11] Arshad A, Atkinson A.J (2022). A 21^st^ century view of the anatomy of the cardiac conduction system. Transl. Res. Anat.

[12] James T.N, Sherf L, Fine G, Morales A.R (1966). Comparative ultrastructure of the sinus node in man and dog. Circulation.

[13] Kawashima T, Sato F (2021). First *in situ* 3D visualization of the human cardiac conduction system and its transformation associated with heart contour and inclination. Sci. Rep.

[14] Thiyagarajah A, Lau D.H, Sanders P (2018). Atrial fibrillation and conduction system disease:The roles of catheter ablation and permanent pacing. J. Interv. Card. Electrophysiol.

[15] Johnson M.S, Martin M.W.S, Henley W (2007). Results of pacemaker implantation in 104 dogs. J. Small. Anim. Pract.

[16] Visser L.C, Keene B.W, Mathews K.G, Browne W.J, Chanoit G (2013). Outcomes and complications associated with epicardial pacemakers in 28 dogs and 5 cats. Vet. Surg.

[17] Peters C.H, Sharpe E.J, Proenza C (2020). Cardiac pacemaker activity and aging. Annu. Rev Physiol.

[18] Logantha S.J.R.J, Yamanushi T.T, Absi M, Temple I.P, Kabuto H, Hirakawa E, Quigley G, Zhang X, Gurney A.M, Hart G, Zhang H, Dobrzynski H, Boyett M.R, Yanni J (2023). Remodelling and dysfunction of the sinus node in pulmonary arterial hypertension. Phil. Trans. R. Soc. B.

[19] Ward J.L, DeFrancesco T.C, Tou S.P, Atkins C.E, Griffith E.H, Keene B.W (2016). Outcome and survival in canine sick sinus syndrome and sinus node dysfunction:93 cases (2002–2014). J. Vet. Cardiol.

[20] DeLay J (2017). Postmortem findings in Ontario racehorses, 2003–2015. J. Vet. Diagn. Invest.

[21] Boden L.A, Charles J.A, Slocombe R.F, Sandy J.R, Finnin P.J, Morton J.M, Clarke A.F (2005). Sudden death in racing thoroughbreds in Victoria, Australia. Equine. Vet. J.

[22] Diab S.S, Poppenga R, Uzal F.A (2017). Sudden death in racehorses:Postmortem examination protocol. J. Vet. Diagn. Invest.

[23] Kiryu K, Nakamura T, Kaneko M, Oikawa M, Yoshihara T (1987). Cardiopathology of sudden cardiac death in the racehorse. Heart Vessels Suppl.

[24] Lyle C.H, Uzal F.A, McGorum B.C, Aida H, Blissitt K.J, Case J.T, Charles J.T, Gardner I, Horadagoda N, Kusano K, Lam K, Pack J.D, Parkin T.D, Slocombe R.F, Stewart B.D, Boden L.A (2011). Sudden death in racing Thoroughbred horses:An international multicentre study of post mortemfindings. Equine. Vet. J.

[25] De Lange L, van Steenkist G, Vera L, de Clerq D, Decloedt A, Cromheeke K.M.C, van Loon G (2019). First Successful Application of a Closed Loop Stimulation Pacemakers with Remote Monitoring in Two Syncopal Miniature Donkeys.

[26] James T.N (1964). Anatomy of the AV node of the dog. Anal. Rec.

[27] Denes P, Wu D, Dhingra R, Amat-y-Leon F, Wyndham C, Rosen K.M (1975). Dual atrioventricular nodal pathways. A common electrophysiological response. Br. Heart J.

[28] De Almeida M.C, Sanchez-Quintana D, Davis N, Charles F.R, Chikweto A, Sylvester W, Loukas M, Anderson R.H (2020). The Ox atrioventricular conduction axis compared to human in relation to the original investigation of Sunao Tawara. Clin. Anat.

[29] Moe G.K, Cohen W, Vick R.L (1963). Experimentally induced paroxysmal AV nodal tachycardia in the dog. A “case report”. Am. Heart. J.

[30] Cabrera J.A, Anderson R.H, Macias Y, Nevado-Medina J, Porta-Sanchez A, Rubio J.M, Sanchez-Quintana D (2020). Variable arrangement of the atrioventricular conduction axis within the triangle of Koch:Implications for permanent His bundle pacing. JACC. Clin. Electrophysiol.

[31] Wilson C, Zi M, Smith M, Hussain M, D´Souza A, Dobrzynski H, Boyett M.R (2023). Atrioventricular node dysfunction in pressure overload-induced heart failure-Involvement of the immune system and transcriptomic remodelling. Front. Pharmacol.

[32] Lo H.M, Lin F.Y, Cheng J.J, Tseng Y.Z (1995). Anatomic substrate of the experimentally-created atrioventricular node re-entrant tachycardia in the dog. Int. J. Cardiol.

[33] Kay G.N, Epstein A.E, Dailey S.M, Plumb V.J (1992). Selective radiofrequency ablation of the slow pathway for the treatment of atrioventricular nodal re-entrant tachycardia. Evidence for involvement of perinodal myocardium within the re-entrant circuit. Circulation.

[34] Kaneshige T, Machida N, Yamamoto S, Nakao S, Yamane Y (2007). A histological study of the cardiac conduction system in canine cases of mitral valve endocardiosis with complete atrioventricular block. J. Comp. Path.

[35] Gómez-Torres F.A, Ruíz-Saurí A (2021). Morphometric analysis of the His bundle (atrioventricular fascicle) in humans and other animal species. Histological and immunohistochemical study. Vet. Res. Commun.

[36] Kalyanasundaram A, Li N, Hansen B.J, Zhao J, Fedorov V.V (2019). Canine and human sinoatrial node:Differences and similarities in the structure, function, molecular profiles, and arrhythmia. J. Vet. Cardiol.

[37] Machida N, Hirakawa A (2021). The anatomical substrate for sick sinus syndrome in dogs. J. Comp. Path.

[38] Semelka M, Gera J, Usman S (2013). Sick sinus syndrome:A review. Am. Fam. Physician.

[39] Demoulin J.C, Kulbertus H.E (1978). Histopathological correlates of sinoatrial disease. Br. J. Cardiol.

[40] Glukhov A.V, Hage L.T, Hansen B.J, Pedraza-Toscano A, Vargas-Pinto P, Hamlin R.L, Weiss R, Carnes C.A, Billman G.E, Fedorov V.V (2013). Sinoatrial node re-entry in a canine chronic left ventricular infarct model:Role of intranodal fibrosis and heterogeneity of refractoriness. Circ. Arrhythm. Electrophysiol.

[41] Monfredi O, Boyett M.R (2015). Sick sinus syndrome and atrial fibrillation in older persons-a view from the sinoatrial nodal myocyte. J. Mol. Cell. Cardiol.

[42] Nakao S, Hirakawa A, Fukushima R, Kobayashi M, Machida N (2012). The anatomical basis of bradycardia-tachycardia syndrome in elderly dogs with chronic degenerative valvular disease. J. Comp. Pathol.

[43] Hansen B.J, Zhao J, Csepe T.A, Moore B.T, Li N, Jayne L.A, Kalyanasundaram A, Lim P, Bratasz A, Powell K.A, Simonetti O.P, Higgins R.S.D, Kilic A, Mohler P.J, Janssen P.M.L, Weiss R, Hummel J.D, Fedorov V.V (2015). Atrial fibrillation driven by micro-anatomic intramural re-entry revealed by simultaneous sub-epicardial and sub endocardial optical mapping in explanted human hearts. Eur. Heart. J.

[44] Van Loon G, Fonteyne W, Rottiers H, Tavernier R, Deprez P (2002). Implantation of a dual-chamber, rate-adaptive pacemaker in a horse with suspected sick sinus syndrome. Vet. Rec.

[45] Keen J.A (2020). Pathological bradyarrhythmia in horses. Vet. J.

[46] Holmes J.R (1987). Heart block in the horse. Equine Cardiology., editor. Cardiac Rhythm.

[47] Liu S.K, Hsu F.S, Lee R.C.T (1989). Diseases of conduction system. An Atlas of Cardiovascular Pathology.

[48] Ertelt A, Bertram C.A, Zuraw A, Gehlen H (2018). A third degree AV-block in a horse with a putative regular rhythm and physiological heart rate in general examination. Pferdeheilkunde Equine Med.

[49] Marolf V, Mirra A, Fouché N, de Solis C.N (2018). Advanced atrioventricular blocks in a foal undergoing surgical bladder repair:First step to cardiac arrest?. Front. Vet. Sci.

[50] Van Loon G (2019). Cardiac arrhythmias in horses. Vet. Clin. North. Am. Equine Pract.

[51] Yoshimura A, Yamaguchi T, Kawazato H, Takahashi N, Shimada T (2014). Immunohistochemistry and three-dimensional architecture of the intermediate filaments in Purkinje cells in mammalian he arts. Med. Mol. Morphol.

[52] Nooma K, Saga T, Iwanaga J, Tabira Y, Watanabe K, Tubbs R.S, Yamaki K.I (2020). A novel method with which to visualize the human sinuatrial node:Application for a better understanding of the gross anatomy of this part of the conduction system. Clin Anat.

